# The effects of weight-bearing manipulations on gait and its underlying neural control mechanisms in toe walking children

**DOI:** 10.3389/fnhum.2025.1701454

**Published:** 2025-11-10

**Authors:** Michelle Gwerder, Rosa M. S. Visscher, Anusha Spescha, Seyyed H. Hosseini Nasab, Yong K. Kim, Regine Zibold, Reinald Brunner, William R. Taylor, Elke Viehweger, Navrag B. Singh

**Affiliations:** 1 Laboratory for Movement Biomechanics, Department of Health Sciences and Technology, ETH Zürich, Zürich, Switzerland; 2 Department of Biomedical Engineering, University of Basel, Basel, Switzerland; 3 Centre for Clinical Motion Analysis, University Children's Hospital Basel, Basel, Switzerland; 4 Department of Orthopaedics, University Children's Hospital Basel, Basel, Switzerland; 5 Singapore-ETH Centre, Future Health Technologies Program, CREATE Campus, Singapore, Singapore

**Keywords:** toe walking, dynamic stability, margin of stability, H-reflex, neuromotor control, weight loading and unloading

## Abstract

**Introduction:**

In toe walking children, impaired maturation of neuromotor control often leads to persistent use of immature motor programs. Understanding the underlying etiology of toe walking in children with cerebral palsy (CP) and idiopathic toe walking (ITW) is crucial for advancing rehabilitation strategies. This study examined gait adaptations and H-reflex responses to varied weight-bearing conditions to determine whether children with ITW and CP exhibit distinct neuromotor control strategies compared to typically developing (TD) peers.

**Methods:**

Eight children with CP (mean age 12.9 ± 2.1 years), eight with ITW (8.6 ± 1.9 years), and 19 TD children (10.0 ± 2.6 years) walked on a treadmill under three conditions: normal bodyweight, 30% bodyweight unloading, and 30% additional bodyweight. Linear mixed-effects models assessed spatiotemporal gait parameters, margin of stability, gait variability, and H-reflex responses.

**Results:**

Bodyweight unloading increased single-limb support time, while reducing double-limb support time and antero-posterior margin of stability across groups (*p* < 0.01). ITW children exhibited increased gait variability (*p* < 0.01) under bodyweight unloading, while CP children showed no change. H-reflex amplitudes decreased under bodyweight unloading in TD children, while CP children exhibited hyperreflexia (*p* < 0.05).

**Discussion:**

The findings of this exploratory study suggest that toe walking is associated with distinct adaptive strategies in ITW and CP children to compensate for environmental challenges. In ITW, increased variability under bodyweight unloading may reflect exploratory motor control, whereas CP children relied on stiffening strategies, marked by reduced variability and hyperreflexia, indicating limited adaptability and less efficient gait patterns. These results imply that similar biomechanical constraints evoke divergent neuromotor adaptations in ITW and CP children.

## 1 Introduction

Walking is the most fundamental form of human locomotion, essential for autonomy, wellbeing, and active participation in daily life. During early gait development, walking is initially unstable and highly variable. Novice walkers make initial floor contact with a flatfoot pattern (Alvarez et al., 2008) before gradually developing a heel strike within the first year of independent walking, enhancing gait stability and efficiency (Hallemans et al., 2006; Zeininger et al., 2018). This developmental shift relies on complex neuromotor adaptations, including the maturation and proper control of muscle reflexes, which play a critical role in supporting posture and coordinating movement during walking (Geyer and Herr, 2010; Ivanenko and Gurfinkel, 2018). However, some children do not develop a heel strike pattern, resulting in persistent toe walking, which may indicate an underlying neurodevelopmental pathology. The most common neurodevelopmental disorder in early childhood is cerebral palsy (CP) (Cans et al., 2004), with an estimated prevalence of 1.6 per 1000 live births (McIntyre et al., 2022). Here, lesions in the central nervous system could arise from damage to the fetal or infant brain (Rosenbaum et al., 2007), leading to reduced supra-spinal involvement in gait adaptation and regulation (Cappellini et al., 2020). Impaired supra-spinal and spinal control mechanisms lead to irregular locomotion, frequently resulting in persistent toe walking patterns, which affect balance and increase the risk of falling (Schlough et al., 2020).

When CP and other medical causes of toe walking are excluded, the pattern is classified as idiopathic toe walking (ITW). ITW affects up to 5% of children (Engstrom and Tedroff, 2012) and is often associated with developmental delays (Shulman et al., 1997), yet its underlying etiology remains unclear. In both CP and ITW, toe walking has been linked to impaired proprioception, motor control difficulties, balance deficits, reduced ankle range of motion, shortened calf muscles, increased energy expenditure, and a heightened risk of falls (Ruzbarsky et al., 2016; De Oliveira et al., 2021; Boyer and Patterson, 2018). In children with CP, spasticity characterized by increased muscle tone and hyperreflexia is a common contributor to abnormal gait patterns. Although hyperreflexia is difficult to quantify through standard motion analysis, it remains a key target in clinical management. Current treatments, such as physiotherapy, orthoses, Botox injections, and surgery, all primarily address the symptoms rather than the underlying causes (Bauer et al., 2022; Novak et al., 2020). However, toe walking and balance issues often persist despite intervention (van Kuijk et al., 2014), leading to frustration for patients and families when treatments fail to provide lasting improvements (Bartoletta et al., 2021; Gillani et al., 2021). A deeper understanding of the neuromotor etiology underlying toe walking is essential before targeted therapies that enhance stability, mobility, and quality of life for affected children can be developed.

Dynamic stability during walking can be assessed using the inverted pendulum model, which assumes balance is maintained when the center of mass (CoM) remains within the base of support (BoS) (Bruijn and van Dieen, 2018; Winter, 1995). The margin of stability (MoS), defined as the distance between the extrapolated CoM (XCoM) and the center of pressure (CoP), is a commonly used measure to assess dynamic stability during walking (Hof et al., 2005). When CoP data are unavailable, foot markers can approximate BoS boundaries (Hof et al., 2005; Watson et al., 2021; Curtze et al., 2024). This metric provides valuable insights into walking strategies and fall risk. However, interpretation of MoS can be complex and paradoxical. A common interpretation is that individuals with reduced stability adopt more cautious gait patterns. Children with ITW often display lower MoS, suggesting a reliance on a falling forward strategy to maintain momentum (Gwerder et al., 2025a), which may serve as a compensatory mechanism for greater energy absorption at initial contact (Gwerder et al., 2025a). Conversely, the opposite relationship can also occur. Stable individuals may afford seemingly unstable gait patterns, while those with poor stability adopt more cautious and stable gait patterns (Sangeux et al., 2024). In typically developing (TD) children, for example, MoS decreases with age, reflecting improved control of the CoM (Ivanenko et al., 2007; Hallemans et al., 2018). Children with CP, in contrast, exhibit impaired pendular energy transmission during gait (van den Hecke et al., 2007), often resulting in increased MoS as a strategy to enhance stability within the limits of reduced motor coordination (Rethwilm et al., 2021; Tracy et al., 2019). These findings underscore the complexity of interpreting MoS solely in terms of stability and further emphasize that gait is a flexible, adaptive process shaped by both biomechanical constraints and neuromotor control strategies.

In early stages of motor development, proprioceptive and visual feedback mechanisms used for gait adaptation are incomplete, indicating immature neuronal feedback systems (Berger et al., 1985). At this stage, motor programs are still undergoing refinement, characterized by higher gait variability, excessive muscle co-activation and strong reflex responses (Berger et al., 1985). With experience and practice, both supra-spinal and spinal control improve, reducing gait variability and enhancing walking efficiency, but also providing high level control to avoid environmental challenges (Ivanenko et al., 2007). In addition to voluntary motor control, reflex responses play a crucial role in gait adaptation. One approach to probe spinal control and neural excitability is the electrically triggered short latency Hoffmann-reflex (H-reflex), which provides insights into spinal circuit function and neuromuscular adaptations (Ivanenko and Gurfinkel, 2018; Theodosiadou et al., 2023). Its amplitude is known to vary with postural demands, the phase of the gait cycle, and different weight-bearing conditions (Capaday and Stein, 1986; Cecen et al., 2018; Angulo-Kinzler et al., 1998; Kim et al., 2024). In TD children, the maturation process is reflected in the progressive suppression of the H-reflex during walking, suggesting increasing corticospinal tract (CST) involvement and greater central inhibition (Hodapp et al., 2007a). In contrast, children with CP exhibit reduced somatosensory integration and impaired maturation of feedforward processes (Berger et al., 1985; Lorentzen et al., 2019), resulting in a continued reliance on immature motor programs with excessive co-contraction of agonist and antagonist muscles (Lorentzen et al., 2019). Although the H-reflex is rhythmically modulated during gait, its overall suppression is impaired in CP children, resembling spinal control patterns observed in toddlers before CST maturation (Hodapp et al., 2007a). Similarly, children with ITW may exhibit immature supra-spinal control, leading to altered neuromotor control strategies and increased but uncoordinated muscular contractures (Veerkamp et al., 2023). Importantly, atypical H-reflex activity has also been reported in ITW children in prone position (Rossi et al., 2018). Most studies investigating reflex control in toe walking children have been conducted under static conditions (Rossi et al., 2018; Leonard et al., 1990; Marzbani et al., 2016), offering only limited insights into how these mechanisms operate during walking and thus contribute to dynamic balance control. However, dynamic tasks such as walking introduce additional stability demands not captured in static assessments and therefore warrant investigation.

Despite evidence that children with ITW and CP rely on altered motor control strategies, the interplay between reflex modulation, dynamic stability, and gait variability, particularly under challenging environmental demands, remains poorly understood. Although they both suffer from toe walking, the underlying reasons might not be identical, and the H-reflex may serve as a tool to interrogate distinct neuromotor control strategies. Moreover, walking under different weight-bearing conditions provides a unique window into how the neuromotor system adapts to environmental challenges. By examining spatiotemporal gait parameters, dynamic stability, and H-reflex modulation during treadmill walking under different weight-bearing conditions, this study aims to clarify whether TD, ITW, and CP children employ distinct movement strategies and reflex responses to adapt to environmental challenges. These insights may contribute to the design of more effective, individualized rehabilitation strategies for children who exhibit toe walking patterns.

## 2 Materials and methods

### 2.1 Participants

This exploratory study was conducted in accordance with the Declaration of Helsinki and received approval from the Zurich cantonal ethics committee (BASEC-No 2019-00678). All participants were provided with detailed verbal and written explanations of the measurement process prior to participation in the study. For children, written informed consent was obtained from legal guardians, while those aged 14 years and older additionally provided their own consent before participation.

Nineteen TD children, eight with ITW, and eight with mild CP (spastic hemiparesis, GMFCS levels I and II, aged 6–18 years, participated in this study (Table 1). Exclusion criteria were (a) any acute or chronic neurological or musculoskeletal conditions other than ITW or CP, (b) injuries or surgeries affecting the lower extremities within the past year, (c) Botox treatment within the previous 6 months, (d) acute pain, (e) vertigo or balance problems, (f) unable to walk on a treadmill for 4 min, or (g) use of medication that could affect balance.

**Table 1:** Anthropometric data.

**Parameter**	**TD**	**ITW**	**CP**
Sample size [*n*]	19	8	8
Gender	9 M, 10 F	5 M, 3 F	4 M, 4 F
Age [years]	10.0 ± 2.6	8.6 ± 1.9	12.9 ± 2.1
Height [cm]	135.9 ± 35.3	134.3 ± 11.7	152.7 ± 11.3
Mass [kg]	36.5 ± 12.0	28.3 ± 7.0	43.6 ± 7.7
Walking speed [m/s]	0.83 ± 0.13	0.75 ± 0.21	0.71 ± 0.20
GMFCS level	–	–	7 I, 1 II

Means ± SD of the three groups: TD, Typically developing; ITW, idiopathic toe walking; CP, cerebral palsy children.

### 2.2 Experimental setup and procedure

#### 2.2.1 Overview

Prior to measurement, the optimal location for eliciting an H-reflex response was marked (see details under 3.2.2. H-reflex). After electrode placement and the attachment of 62 whole-body reflective markers (Supplementary material, 1. Marker Set), individualized stimulus intensity was established using H-reflex recruitment curves (RC, see details under 3.2.2. H-reflex) while participants walked barefoot on a treadmill (h/p/cosmos quasar, h/p/cosmos sports & medical GmbH, Germany). Kinematic data were recorded at 200 Hz using a 10-camera optical motion-capture system (VICON Motion Systems Ltd, Oxford, UK). At the start of the experiment, each participant familiarized themself with treadmill walking while wearing a safety harness and selected a comfortable walking speed, which was maintained across all conditions.

**Figure 1:**
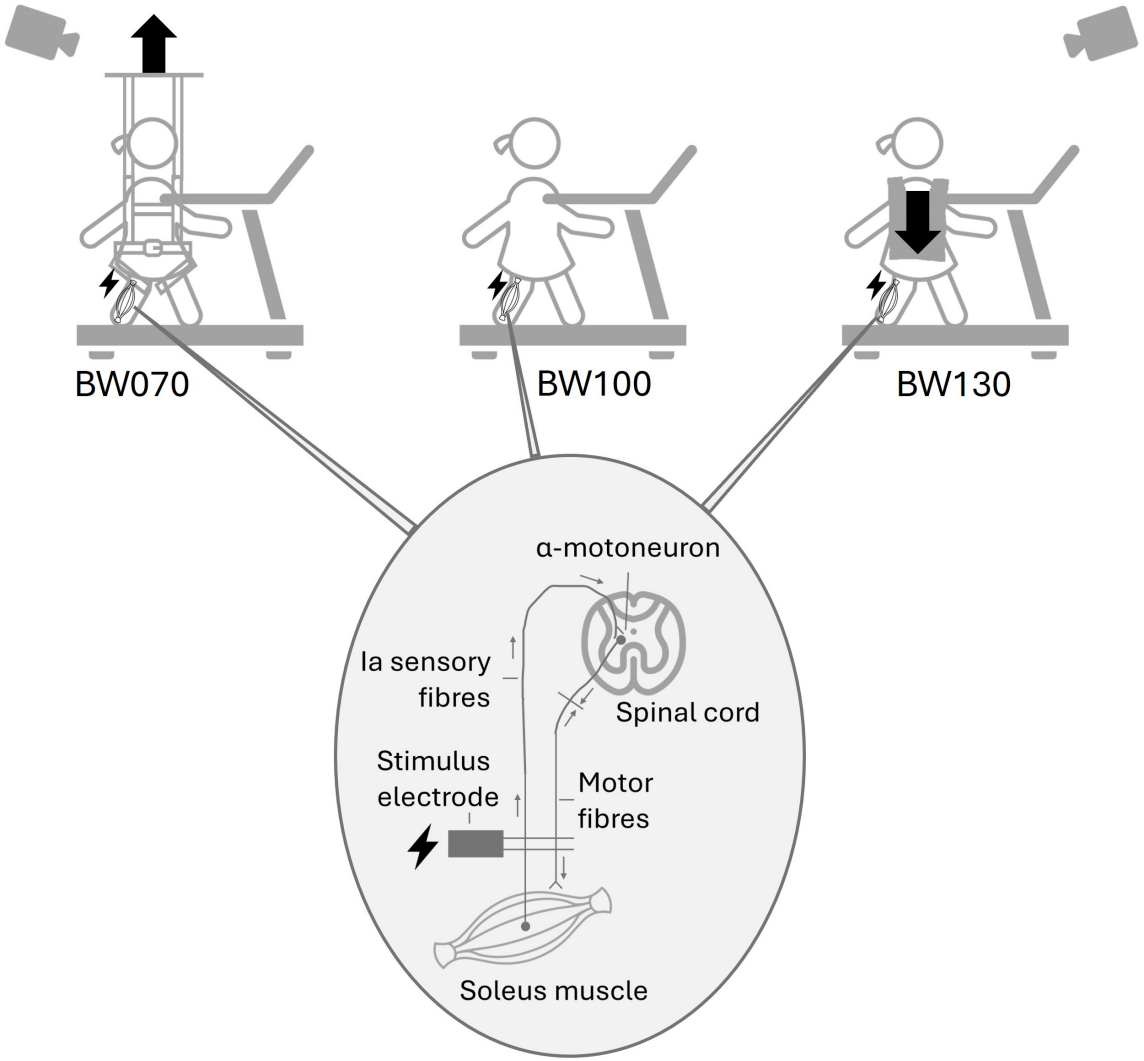
Study setup: Children walked barefoot on a treadmill under three different weight-bearing conditions: normal bodyweight (BW100), 30% bodyweight unloading (BW070), achieved using a bodyweight support system, and 30% additional bodyweight (BW130), applied using a weight vest. Below, the H-reflex mechanism is illustrated, showing the stimulus electrode triggering the nerve in the back of the knee, the Ia sensory fibers connecting with the α-motoneuron in the spinal cord, projecting to the soleus muscle fibers, adapted from Gwerder et al. (2025b).

Three weight-bearing conditions were tested in a randomized order, with each lasting 4 min: 1) baseline condition with normal bodyweight (BW100), 2) 30% bodyweight unloading (BW070) achieved using a bodyweight support system (Ergolet Walking Sling, Winncare Nordic ApS, DK), and c) 30% bodyweight loading (BW130) applied using a weight vest (EZ Vest Pro, Kensui LLC, US) (Figure 1).

#### 2.2.2 H-reflex

The H-reflex was electrically elicited in the non-dominant leg for TD children, while for ITW and CP children, stimulation was applied to the more affected leg. The dominant leg was chosen by asking participants: “If you were kicking a ball, which leg would strike the ball?” (van Melick et al., 2017). A wireless electromyography (EMG) electrode (PicoEMG, Cometa, Italy, 3,000 Hz sampling frequency) was positioned on the soleus muscle. The tibial nerve was then identified in the popliteal fossa in a prone position using a movable electrode, with a rectangular pulse (1ms duration) delivered by a constant current stimulator (DS7AH, Digitimer, UK) (Kim et al., 2024; Gwerder et al., 2025b). Once an H-reflex was successfully elicited (visible H-wave in soleus EMG signal), a self-adhesive stimulation cathode (Ag/AgCl Kendall™ H124SG, CardinalHealth, Switzerland) was fixed at the location identified by the movable electrode.

To standardize the H-reflex measurement, optimal stimulation intensity was firstly determined by quantifying the subject-specific H-reflex recruitment curve (RC) during walking (Kim et al., 2024; Gwerder et al., 2025b). Here, a series of stimuli were delivered between heel strike and mid-stance (identified as the gait phase most sensitive to H-reflex modulation Chalmers and Knutzen, 2002; Krauss and Misiaszek, 2007 using MATLAB (R2018a, The MathWorks, Inc., Natick, United States; RRID:SCR_001622). The stimulus intensity was increased from 10 to 15 mA in increments of approximately 1 mA until the M-wave peak-to-peak amplitude reached a plateau (M_max_) (Palmieri et al., 2004). Due to discomfort or pain, it was not possible to reach M_max_ for every participant during the stimulation procedure, whereby the stimulation was stopped at the point of discomfort. Gaussian and sigmoid functions were fitted to the data to extract peak-to-peak amplitudes of H-reflex and the associated M-wave where possible. Optimal stimulus intensity was then standardized to the intensity corresponding to 80% of each participant's individual maximum H-reflex amplitude. This intensity was chosen because it reliably produces a sufficiently large and reproducible reflex response that also remains sensitive to changes, while minimizing the risk of antidromic collision with the M-wave since this intensity can be identified on the ascending phase of the H-reflex curve (Kim et al., 2024; Palmieri et al., 2004; Crone et al., 1990; Bouguetoch et al., 2020).

Finally, patients were tested over a period of 4 min of treadmill walking for each weight-bearing condition. Throughout the measurement period, electrical stimulation to elicit an H-reflex was applied approximately every 9–16 s to avoid familiarization (Chen and Zhou, 2011), and was delivered between heel strike and mid-stance similar to the procedures used to determine the optimal stimulation intensity. Marker kinematic data and EMG signals were measured to determine gait events, dynamic stability parameters, and peak-to-peak reflex responses.

### 2.3 Data analysis

To ensure analysis of steady-state walking, the first and last 30 s of each 4-min walking trial were excluded to avoid transients. Gait events were detected based on kinematic data from foot markers, using a validated algorithm shown to have high accuracy in identifying gait patterns in toe walking children (Ghoussayni et al., 2004; Visscher et al., 2021). The spatiotemporal gait parameters evaluated from each stride included: stride time, single- and double-limb support time, step width, and stride length. The mean and standard deviation (SD) were calculated, with SD serving as a measure of gait variability. Spatiotemporal parameters were analyzed for the stimulated leg only.

#### 2.3.1 Dynamic stability

To assess dynamic stability, the margin of stability (MoS) was calculated in both the antero-posterior (MoS_AP_) and medio-lateral (MoS_ML_) directions (Hof et al., 2005). To support an accurate CoM estimation in toe walking children, an OpenSim model (OpenSim v4.3, RRID:SCR_002683) was additionally constructed that included all segment kinematics. Here, the model was scaled to represent each participant's anthropometry using marker trajectories captured during a standing trial, while a linear scaling method was used to estimate each segment's mass based on the overall bodyweight. Inverse and body kinematics tools were used to compute the CoM and extrapolated CoM (XCoM) throughout the measured trials:


$$\begin{array}{c} XCoM=CoM+\;\frac{CoM_{v}}{\sqrt{\frac{g}{l}}} \end{array}$$

where *CoM*_*v*_ was the velocity of the *CoM* adjusted for treadmill walking, *g* the gravitational constant $g=9.81\;\frac{m}{s^{2}}$ and *l* the length from the *CoM* to the midpoint between the two malleoli markers.

The MoS was determined at heel strike, as this moment dictates foot placement for the stance phase (Hof, 2008) and is important to maintain balance (Wallard et al., 2014). The boundaries of the base of support (*BoS*) were defined using the first metatarsal marker for antero-posterior and the fifth metatarsal marker for the medio-lateral limits:


$$\begin{array}{c} MoS=BoS-XCoM \end{array}$$

Note that this approach results in an overestimation of the MoS compared to methods that use the CoP (Hof and Curtze, 2016).

#### 2.3.2 H-reflex

**Figure 2:**
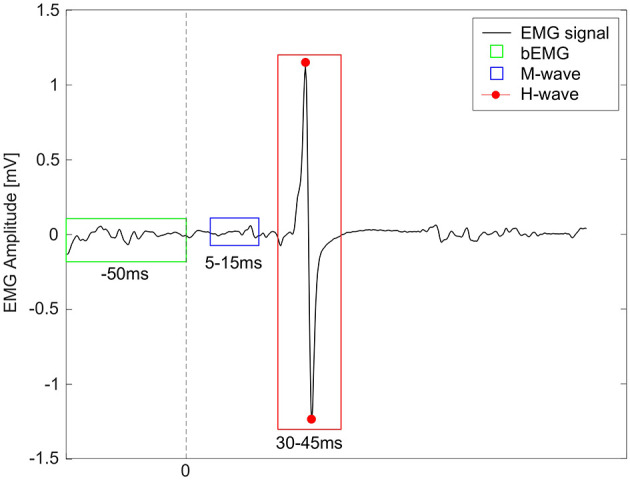
Example of a soleus EMG signal (black) from a child with idiopathic toe walking. The dashed line at 0 represents the timing of the electrical stimulus. The green box represents the window for the baseline EMG (bEMG), the blue box for the M-wave, and the red box shows the peak-to-peak amplitude of the H-reflex (highlighted with red points).

For each participant and condition, average peak-to-peak amplitudes of the H-reflex (30–45 ms after stimulus) were calculated, based on average 14 stimuli per condition (see Figure 2 for example). Some stimulations fell outside early stance phase, mainly due to marker occlusion issues, and were hence excluded (Simonsen and Dyhre-Poulsen, 1999). Since it was not possible to identify an M_max_ value for each child from the RCs, but to still ensure consistency and comparability, the inclusion of H-reflex responses was dependent upon stable muscle activity during the M-wave time window. Specifically, the EMG amplitude within this window was required to fall within ± 20% of the condition-specific mean (Kim et al., 2024; Gwerder et al., 2025b). Additionally, H-reflex amplitudes were normalized (H-reflex_norm_) to average baseline EMG (bEMG) activity, assessed 50 ms before each stimulation, but after heel strike (Palmieri et al., 2004).

### 2.4 Statistical analysis

Linear mixed-effects models were used to analyse gait parameters, gait variability, and H-reflex adaptations across different weight-bearing conditions and groups. Dependent variables included mean and variability measures of stride time, single- and double-limb support time, stride length, step width, MoS_AP_, and MoS_ML_, as well as H-reflex amplitude, H-reflex_norm_, and bEMG. Group (TD, ITW, and CP) and bodyweight condition (BW070, BW100, BW130) were treated as fixed effects, along with their interaction, while participants were included as random effects in each model. BW100 served as the baseline for all comparisons. Statistical significance was set at *p* ≤ 0.05 and adjusted for multiple comparisons where appropriate using the false discovery rate method (Benjamini and Hochberg, 1995). All analyses were conducted in RStudio (v2024.04.2, R Core Team, Austria, RRID:SCR_000432).

## 3 Results

### 3.1 Gait adaptations

**Figure 3:**
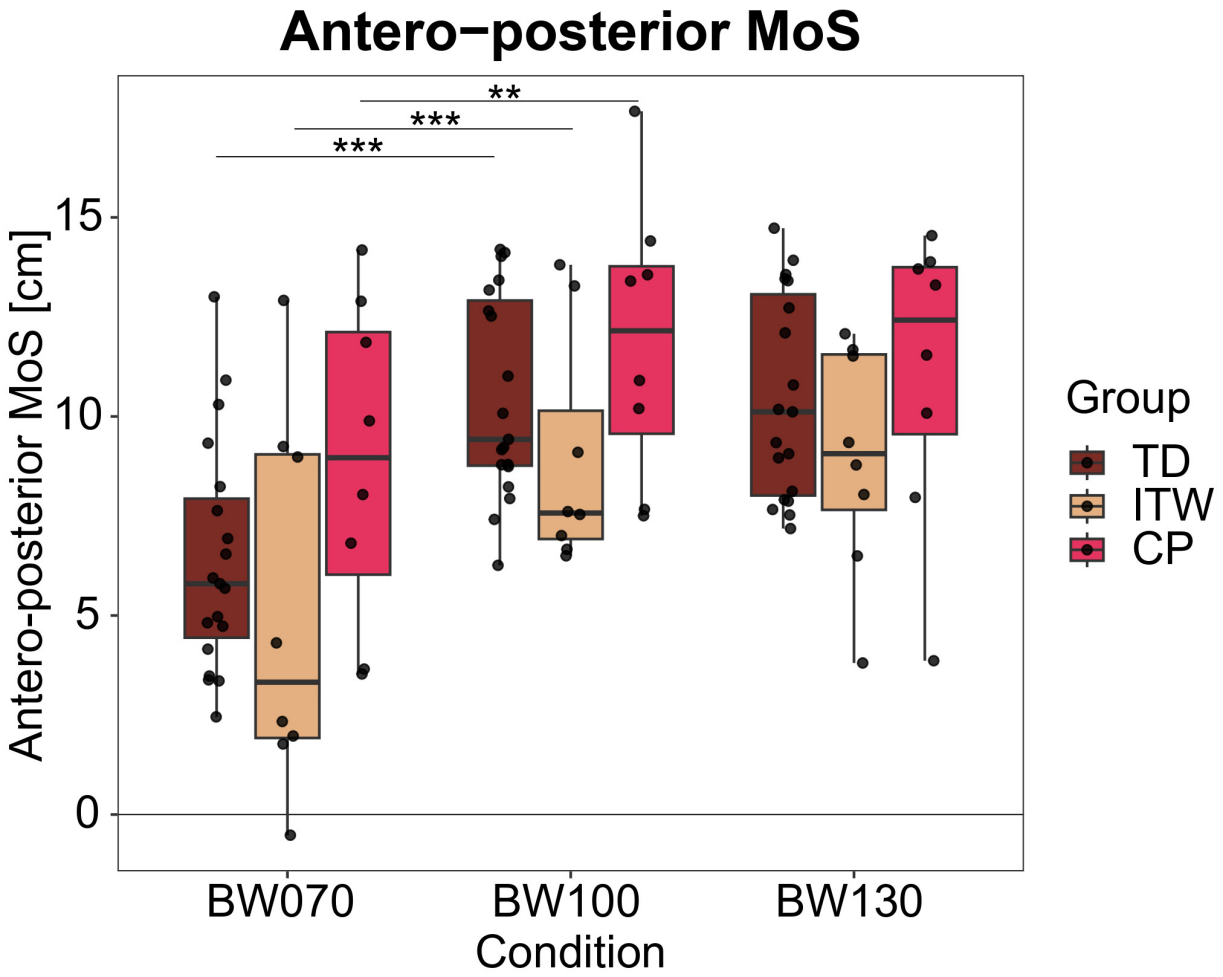
Antero-posterior margin of stability (MoS) for the three different weight-bearing conditions normal bodyweight (BW100), 30% bodyweight unloading (BW070), and 30% additional bodyweight (BW130) for typically developing (TD, dark red), idiopathic toe walking (ITW, orange), and cerebral palsy (CP, pink) children. ** (*p* < 0.01), *** (*p* < 0.001) show significant differences from baseline (BW100).

An average of 145 strides per participant and condition was analyzed. Under the BW070 condition, single-limb support time significantly increased (*p* < 0.001, Table 2 and Supplementary material, 2. Statistical Analysis Table S1) by 8% in TD, 13% in ITW, and 7% in CP children compared to BW100. On the other hand, double-limb support time decreased by 19% in TD, 10% in ITW, and 15% in CP children respectively. CP children exhibited significantly increased double-limb support time compared to TD children (*p* < 0.001, Table 2 and Supplementary material, 2. Statistical Analysis Table S1). MoS_AP_ was significantly decreased in BW070 by 39% in TD, 43% in ITW, and 26% in CP children compared to BW100 (*p* < 0.01, Figure 3).

**Table 2:** Mean gait parameters.

**Parameter**	**BW070**	**BW100**	**BW130**
**Typically developing**			
Cadence [steps/min]	113.8 ± 11.4	114.1 ± 10.3	116.5 ± 11.6
Stride time [s]	1.07 ± 0.11	1.06 ± 0.09	1.04 ± 0.08
Single support [s]	**0.43** ±0.05^***^	0.40 ± 0.04	0.38 ± 0.03
Double support [s]	**0.21** ±0.06^**^	0.26 ± 0.05	0.27 ± 0.06
Stride length [cm]	97.8 ± 17.1	97.8 ± 17.6	97.1 ± 16.7
Step width [cm]	12.5 ± 2.3	12.2 ± 2.7	12.0± 1.7
MoS_AP_ [cm]	**6.4** ±2.9^***^	10.5 ± 2.6	10.5 ± 2.6
MoS_ML_ [cm]	11.1 ± 1.5	10.4 ± 1.4	10.6 ± 1.3
**Idiopathic toe walking**			
Cadence [steps/min]	113.2 ± 19.6	112.9 ± 15.5	114.0± 13.0
Stride time [s]	1.14 ± 0.20	1.09 ± 0.19	1.08 ± 0.18
Single support [s]	**0.44** ±0.05^***^	0.39 ± 0.04	0.39 ± 0.04
Double support [s]	0.27 ± 0.11	0.30 ± 0.12	0.30 ± 0.12
Stride length [cm]	81.2 ± 17.1	79.0 ± 17.1	78.6 ± 16.2
Step width [cm]	12.5 ± 2.6	11.8 ± 2.4	11.6 ± 2.4
MoS_AP_ [cm]	**5.1** ±4.7^***^	8.9 ± 3.0	9.0± 2.9
MoS_ML_ [cm]	10.4 ± 2.1	9.3 ± 1.4	9.2 ± 1.4
**Cerebral palsy**			
Cadence [steps/min]	102.1 ± 16.1	98.5 ± 20.5	100.4 ± 20.0
Stride time [s]	**1.29** **±0.27†**	**1.29** **±0.31†**	**1.29** **±0.28†**
Single support [s]	**0.45** ±0.06^**^	0.42 ± 0.06	0.43 ± 0.06
Double support [s]	**0.35** ±0.17^**^†	**0.41** **±0.21†**	**0.41** **±0.20†**
Stride length [cm]	88.1 ± 17.6	87.7 ± 14.5	85.4 ± 16.8
Step width [cm]	11.7 ± 1.9	12.4 ± 2.9	11.4 ± 3.6
MoS_AP_ [cm]	**8.9** ±4.0^**^	11.9 ± 3.5	11.1 ± 3.7
MoS_ML_ [cm]	11.0 ± 2.5	11.8 ± 2.2	11.4 ± 1.7

Mean ± SD for the three different weight-bearing conditions in typically developing, idiopathic toe walking, and cerebral palsy children. Pairwise *post-hoc* comparison tests highlighted significant differences from baseline (BW100, ^**^p < 0.01, ^***^p < 0.001) and † denote significant group differences between pathological and typically developing groups (*p* < 0.05). MoS_AP_: antero-posterior margin of stability; MoS_ML_: medio-lateral margin of stability; BW070: 30% bodyweight unloading; BW100: normal bodyweight; BW130: 30% additional bodyweight.

For BW130, no significant differences were found in any groups compared to BW100.

### 3.2 Gait variability

In the BW070 condition, variability in single- and double-limb support times increased significantly in TD children by 50% and 26%, respectively, and in ITW children by 74% and 50% compared to BW100. MoS_AP_ variability also rose significantly, by 7% in TD and 55% in ITW children in the bodyweight unloading condition (*p* < 0.05, Table 3 and Supplementary material, 2. Statistical Analysis Table S2).

**Table 3:** Gait variability parameters.

**Parameter**	**BW070**	**BW100**	**BW130**
**Typically developing**			
Stride time [ms]	52.8 ± 27.8	42.2 ± 23.7	39.6 ± 23.8
Single support [ms]	**26.4** ±13.8^**^	17.6 ± 8.3	15.9 ± 7.2
Double support [ms]	**36.4** ±15.8^*^	28.9 ± 16.1	27.3 ± 14.6
Stride length [cm]	7.1 ± 3.0	6.1 ± 2.6	5.7 ± 2.4
Step width [cm]	3.0 ± 1.4	2.7 ± 1.2	3.3 ± 1.0
MoS_AP_ [cm]	**3.0** ±1.1^*^	2.8 ± 1.2	2.5 ± 0.8
MoS_ML_ [cm]	2.0 ± 0.6	1.7 ± 0.6	1.7 ± 0.5
**Idiopathic toe walking**			
Stride time [ms]	**88.5** ±37.9^**^	56.0 ± 23.4	59.6 ± 24.3
Single support [ms]	**39.6** ±13.0^**^	22.8 ± 10.4	25.0 ± 11.2
Double support [ms]	**60.7** ±21.2^***^	40.4 ± 22.3	38.9 ± 15.7
Stride length [cm]	**9.0** ±2.7^**^	6.1 ± 1.7	6.7 ± 2.4
Step width [cm]	3.0 ± 0.7	2.6 ± 0.9	2.9 ± 1.0
MoS_AP_ [cm]	**4.5** ±1.7^**^	2.9 ± 0.6	3.1 ± 0.9
MoS_ML_ [cm]	**2.5** ±0.6^**^	1.9 ± 0.4	1.9 ± 0.3
**Cerebral palsy**			
Stride time [ms]	59.1 ± 52.5	59.8 ± 36.8	58.2 ± 34.3
Single support [ms]	32.1 ± 27.3	27.9 ± 15.8	29.8 ± 17.5
Double support [ms]	42.9 ± 36.4	47.4 ± 27.6	42.8 ± 26.9
Stride length [cm]	6.3 ± 2.8	6.3 ± 1.7	6.0 ± 1.9
Step width [cm]	2.9 ± 1.1	2.0 ± 0.6	2.6 ± 1.0
MoS_AP_ [cm]	3.3 ± 2.8	3.1 ± 1.5	3.2 ± 1.6
MoS_ML_ [cm]	1.8 ± 0.7	1.9 ± 0.6	1.5 ± 0.4

Mean ± SD for the three different weight-bearing conditions in typically developing, idiopathic toe walking, and cerebral palsy children. Pairwise *post-hoc* comparison tests highlighted significant differences from baseline (BW100, ^*^p < 0.05, ^**^p < 0.01, ^***^p < 0.001). No significant differences were observed between groups. MoS_AP_, anterior-posterior margin of stability; MoS_ML_, mediolateral margin of stability; BW070, 30% bodyweight unloading; BW100, normal bodyweight; BW130, 30% additional bodyweight.

Children with ITW exhibited a substantial increase in variability in the BW070 condition, with at least a 32% rise across all parameters except step width. In contrast, children with CP showed no significant changes in any variability parameters under bodyweight unloading compared to baseline.

For bodyweight loading conditions (BW130), no significant differences in variability were observed compared to baseline for any group. Additionally, no significant differences between groups were observed for gait variability parameters.

### 3.3 H-reflex

Across all groups and conditions, a total of 1,338 reflexes were elicited (757 in TD, 307 in ITW, and 281 in CP). A total of 876 (65%) were included in the analysis and the included stimuli were applied on average at 26% of the gait cycle, or 0.29s ± 0.11s following foot contact.

**Figure 4:**
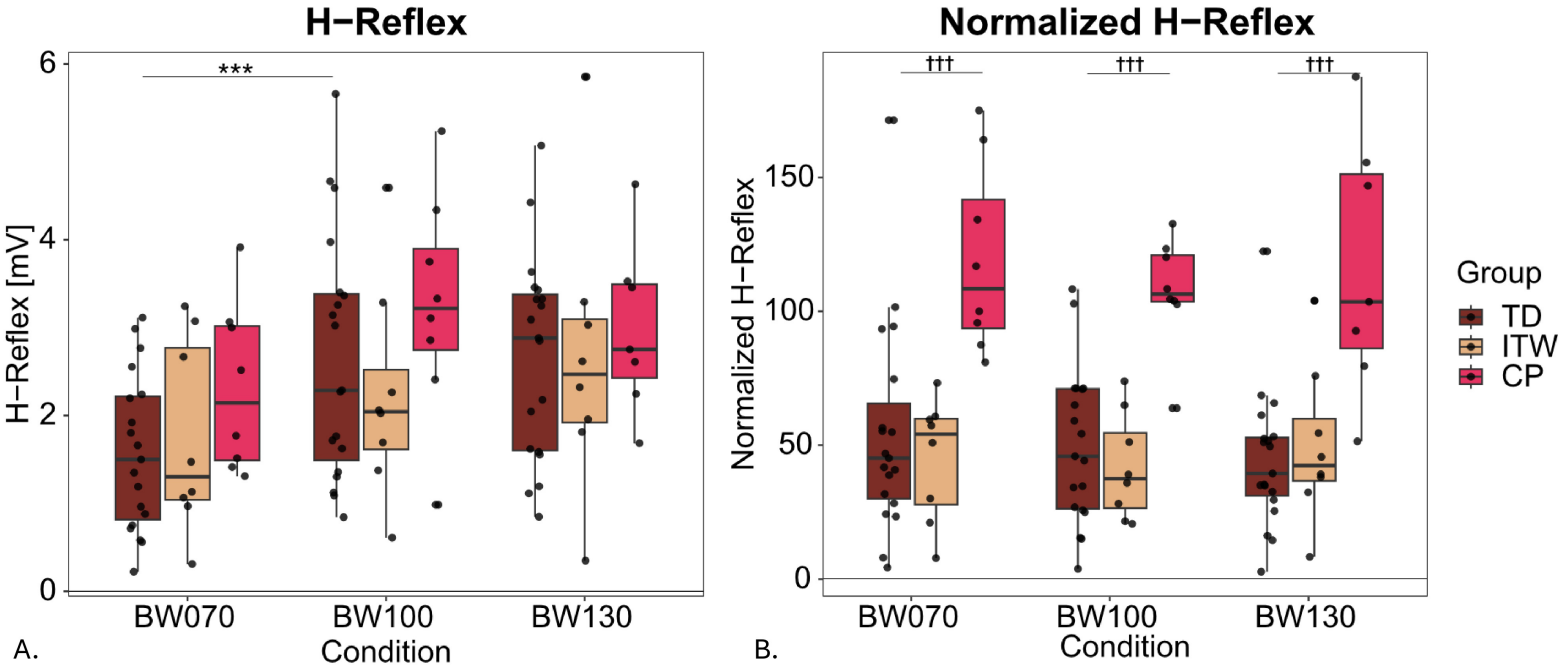
Hofmann reflex (H-reflex) peak-to-peak amplitudes for non-normalized **(A)** and normalized data **(B)** for the three different weight bearing conditions normal bodyweight (BW100), 30% bodyweight unloading (BW070), and 30% additional bodyweight (BW130) for typically developing (TD, dark red), idiopathic toe walking (ITW, orange), and cerebral palsy (CP, pink) children. *** show significant differences from baseline (BW100, *p* < 0.001), while ††† denote significant group differences between pathological and typically developing (TD) groups (*p* < 0.001).

*Post-hoc* comparisons in H-reflex revealed significant decreases for BW070 compared to BW100 in TD children (41%, *p* < 0.001) with similar trends in ITW (22%), and CP (29%, Table 4 and Supplementary material, 2. Statistical Analysis Table S3, Figure 4A). Furthermore, CP children exhibited significant reduced bEMG values compared to TD children (*p* < 0.05). Finally, there was a significant group difference with CP children showing 116% higher H-reflex_norm_ at BW100 compared to TD children (*p* < 0.001, Figure 4B).

**Table 4:** H-reflex, H-reflex_norm_, and baseline EMG (bEMG) activity.

**Parameter**	**BW070**	**BW100**	**BW130**
**Typically developing**			
H-reflex [mV]	**1.58** ±0.88^***^	2.66 ± 1.39	2.68 ± 1.17
H-reflex_norm_	54.45 ± 23.78	49.69 ± 29.04	44.25 ± 25.96
bEMG [mV]	**0.05** ±0.04^*^	0.08 ± 0.06	0.09 ± 0.06
**Idiopathic toe walking**			
H-reflex [mV]	1.74 ± 1.10	2.24 ± 1.22	2.65 ± 1.58
H-reflex_norm_	45.05 ± 22.77	41.87 ± 19.80	49.70 ± 29.13
bEMG [mV]	0.04 ± 0.01	0.06 ± 0.03	0.06 ± 0.01
**Cerebral palsy**			
H-reflex [mV]	2.31 ± 0.96	3.25 ± 1.28	2.99 ± 0.97
H-reflex_norm_	**119.31** **±35.40**^**†††**^	**107.45** **±20.71**^**†††**^	**116.78** **±48.06**^**†††**^
bEMG [mV]	**0.02** **±0.01**^**†**^	**0.03** **±0.01**^**†**^	**0.04** **±0.02**^**†**^

Mean ± SD under the three different weight-bearing conditions in typically developing, idiopathic toe walking, and cerebral palsy children. Pairwise *post-hoc* comparison tests highlighted significant differences from baseline (BW100) (^*^p < 0.05, ^***^p < 0.001), while † denote significant group differences between pathological and typically developing groups (^†^*p* < 0.05, ^†††^*p* < 0.001). BW070: 30% bodyweight unloading; BW100: normal bodyweight; BW130: 30% additional bodyweight.

## 4 Discussion

A deeper understanding of the interplay between dynamic stability, reflex control, and walking strategies in children with ITW and CP is essential for advancing rehabilitation strategies and improving balance, functional mobility, and quality of life. This study aimed to clarify whether TD, ITW, and CP children employ distinct movement strategies and reflex responses to adapt to environmental challenges. Our findings reveal that bodyweight unloading resulted in longer single-limb support time, shorter double-limb support time, and decreased MoS_AP_ in all three cohorts. These changes suggest well-functioning adaptations in temporal gait regulation under challenging environments, and a shift in movement strategy to accommodate the reduced forward momentum during bodyweight unloading. Notably, ITW children exhibited significantly greater variability during bodyweight unloading compared to baseline (BW100), suggesting increased motor exploration to identify an optimal strategy for adapting to gait challenges. Since no baseline differences were observed compared to TD peers, ITW children appeared stable during normal walking but struggled to adjust to the altered unloading condition, leading to increased variability and instability. In contrast, CP children exhibited no significant differences in variability compared to baseline (BW100) for either additional loading or unloading, suggesting either a reliance on a stiffening strategy that persisted even under challenging conditions, or a limited ability to explore alternative motor strategies. One plausible explanation for this lack of adaptation is that children with CP already exhibit impairments and highly individualized movement strategies under normal conditions, which may remain unchanged in the short-term even when facing the additional challenge of bodyweight unloading.

TD children showed a significant reduction in H-reflex amplitude during bodyweight unloading, indicating intact and responsive H-reflex modulation. Children with CP generally exhibited significantly higher H-reflex_norm_ amplitudes compared to TD children. These findings indicate hyperreflexia, or a lack of supra-spinal inhibition in the CP group, possibly suggesting differently developed sensorimotor systems. Interestingly, in ITW children there was no difference in H-reflex_norm_ compared to their TD peers. This result suggests that spinal reflex excitability might be preserved in ITW children. It further indicates that, although their gait pattern is atypical, these children may have developed a stable and well-regulated motor strategy that supports toe walking as a consistent, learned locomotion pattern rather than one primarily driven by abnormal reflex activity. Larger cohort and longitudinal studies from the onset of walking may help to clarify whether this pattern is adaptive or indicative of persistent neurodevelopmental differences.

### 4.1 Mean gait adaptations

This study found that bodyweight unloading resulted in longer single-limb support and shorter double-limb support time compared to baseline across all three groups, indicating an adapted temporal regulation in response to a more challenging environment (Herssens et al., 2021). It is unlikely that unloading in this way has ever been experienced by the participants, resulting in a first-time reaction and immediate change in temporal gait parameters. Similar trends have previously been reported in healthy adults under bodyweight unloading conditions (Kim et al., 2024; Gwerder et al., 2025b; Apte et al., 2018). According to the inverted pendulum model, a reduced double-limb support time limits the duration available for CoM redirection to the next step, restricting opportunities for postural adjustments (Adamczyk and Kuo, 2009). This may contribute to a less efficient, more variable, and potentially unstable gait pattern. Notably, this timing adaptation was consistent across all three groups and did not appear to be significantly influenced by toe walking behavior. Additionally, MoS_AP_ significantly decreased under the bodyweight unloading condition for all groups. A lower MoS_AP_ likely reflects reduced forward momentum, indicating either weaker forward propulsion and diminished push-off forces or a reservation to move into an unfamiliar posture. However, since the harness mitigated any fall possibility but also somewhat restricted forward leaning, it is more likely that an impaired push-off force hindered effective forward projection of the CoM, such that maintenance of stability in the antero-posterior direction remained challenging (Kulkarni et al., 2023).

Trends suggest that ITW children exhibited lower MoS_AP_ compared to TD children across all conditions, while CP children showed an increase. Similar findings have been reported previously (Gwerder et al., 2025a), where a reduced MoS_AP_ in ITW children potentially indicates reliance on an immature, falling forward movement strategy to maintain forward momentum, which includes upper body anchoring to simplify balance control due to a reduced number of degrees of freedom (Gwerder et al., 2025a; Assaiante et al., 2005). Conversely, CP children exhibited the opposite trend, with increased MoS_AP_ compared to TD children, also consistent with previous findings (Sangeux et al., 2024). CP children possibly exhibit impaired pendular energy transmission during gait (van den Hecke et al., 2007). To optimize, they elevate their CoM to improve pendular efficiency and facilitate limb clearance (Zollinger et al., 2016), resulting in increased MoS as a strategy to enhance stability within the limits of reduced motor coordination (Rethwilm et al., 2021; Tracy et al., 2019). This suggests that CP children adopt a more conservative gait strategy, maintaining higher safety margins to compensate for reduced strength, impaired sensorimotor integration, and immature foot trajectory control (Tracy et al., 2019; Zarkou et al., 2020). Due to deficits in intersegmental coordination and muscle activity, they seem to rely on alternative movement strategies with higher safety margins to minimize fall risk as far as possible (Cappellini et al., 2016).

No significant differences were observed under additional bodyweight loading conditions, possibly because children are accustomed to carrying additional weight, such as heavy backpacks. As a result, the added load may not have been sufficiently novel or challenging to elicit changes in gait parameters.

### 4.2 Gait variability

During bodyweight unloading, temporal parameters of gait variability significantly increased in TD children, likely reflecting the greater challenge posed by reduced weight-bearing compared to increased loading. This condition potentially demands a more active gait control strategy (Collins and Kuo, 2013). In novel or demanding environments, the sensorimotor control system often increases variability as an adaptive mechanism, allowing for movement exploration and fine-tuning to meet new stability demands (Abram et al., 2022). Interestingly, no differences were observed in spatial parameters of gait variability in TD children. Why temporal, rather than spatial, aspects of gait variability are specifically adapted under different challenging conditions remains unclear. One possible explanation is that temporal parameters of task performance are more vulnerable to disruption than spatial parameters (Bakir et al., 2013).

TD children exhibited similar responses to healthy adults (Kim et al., 2024; Gwerder et al., 2025b), suggesting effective adaptations to challenging conditions and well-functioning compensatory strategies. Children with ITW showed no differences at baseline, indicating that their gait is well-adapted to normal walking conditions. However, during bodyweight unloading, variability increased across all parameters except step width. This aligns with Fanchiang et al. (2016), who reported greater gait variability in ITW children compared to healthy controls when walking on different surfaces. These findings suggest that although ITW children may have developed a stable walking strategy on level ground, they might prioritize medio-lateral over antero-posterior stability, potentially explaining the preserved step width variability, while exploring alternative movement strategies in other directions when confronted with challenging conditions. For future studies interested in long-term adaptations, it would be valuable to investigate longer exposure durations, as there is some preliminary evidence, that motor control can improve with time in children with ITW (Clark et al., 2010).

In contrast, CP children in our study generally exhibited reduced variability, consistent with a limited repertoire of movements and behavioral strategies (Delcour et al., 2018; Hadders-Algra, 2010). Here, they are thought to rely on a stiffening response with increased muscle co-contractions, producing a more rigid gait pattern when faced with challenges (Lorentzen et al., 2019). However, to fully assess this, future studies should analyse gait variability in combination with muscle activity across several muscles and throughout the entire gait cycle. In the present study, only bEMG immediately preceding the stimulation was examined, which may not accurately reflect overall muscle activation patterns. Additionally, between-subject variability, reflected in the high standard deviations, was notably elevated in CP children. This suggests that while some children exhibited very high others showed very low gait variability, reflecting the heterogeneity in strategies to regulate task performance among various forms of CP. Although the inclusion criteria aimed to create a relatively homogeneous group (spastic hemiparesis and GMFCS levels I and II), individual differences in motor impairments and functional abilities inevitably contributed to the observed variability. Participants were classified in similar GMFCS levels, but heterogeneity in lesion location, muscle tone, prior interventions, and individual differences in strength and motor control likely also influence their movement strategy. These individualized strategies under normal conditions may remain unchanged in the short-term even when facing the additional challenge of bodyweight unloading. Our protocol only provided brief exposure to bodyweight unloading. Our protocol only provided brief exposure to bodyweight unloading, which is likely insufficient to produce neuromotor adaptation in children with mild CP (Cherng et al., 2007). Finally, the absence of variability modification might also indicate the prioritization of stability over flexibility and adaptability, potentially resulting in higher energy expenditure and reflecting the nervous system's limited ability to finely regulate motor output (Delcour et al., 2018; Hak et al., 2013).

### 4.3 H-reflex

Our study observed a significant decrease in H-reflex amplitude under bodyweight unloading in TD children with similar trends in ITW and CP children. This response suggests a healthy regulation of postural control, similar to that observed in young adults (Kim et al., 2024; Gwerder et al., 2025b), and has also been reported during challenging tasks in CP children (Conner et al., 2022). The unfamiliar and challenging nature of bodyweight unloading likely requires greater supra-spinal involvement to maintain stability, shifting motor control away from automatic, reflex-driven adjustments toward more active regulation (Dragunas and Gordon, 2016; Skiadopoulos et al., 2020; Sibley et al., 2007). Additionally, altered sensory feedback (e.g. haptic, proprioceptive) under reduced loading conditions may provide limited support for normal postural control mechanisms, necessitating further active neural adaptation to the situation (Marchant et al., 2020). Given that both ITW and CP children often present with sensory processing difficulties (Williams et al., 2010; Pavao et al., 2015), it is a reasonable assumption that they would be even more affected by these changes. In contrast, bodyweight loading did not significantly alter H-reflex amplitudes in any group. However, children with CP exhibited significantly increased H-reflex_norm_ amplitudes compared to TD children, potentially indicating reduced central inhibition and hyperreflexia (Leonard et al., 1990; Hodapp et al., 2007b). While younger children with CP have shown H-reflex responses comparable to their TD peers in previous research (Cappellini et al., 2016), the age-related increase in reflex suppression typically observed in TD children under normal conditions appears diminished in those with CP, likely reflecting delayed CST maturation (Leonard et al., 1990; Cappellini et al., 2016). Combined with the trend toward reduced gait variability, this suggests that children with CP may have limited capacity and repertoire to explore alternative motor programs, leading to compensatory but less efficient gait strategies and reduced stability. Furthermore, overreliance on reflexive mechanisms is thought to compensate for muscle weakness and poor motor control, assisting lower limb muscles in stabilizing the CoM (Brunner and Götz-Neumann, 2023). This aligns with our findings where CP children exhibited trends toward lower bEMG activity directly before the stimulus, a well-documented issue in CP (Thompson et al., 2011). Thus, increased H-reflex_norm_ activity in CP children may represent an adopted strategy to counteract instability under different weight-bearing conditions.

In contrast to the hyperreflexia observed in children with CP, those with ITW displayed H-reflex responses similarly to their TD peers, although without the expected modulation. Maybe the upper body anchoring to simplify balance control due to a reduced number of degrees of freedom also limits H-reflex modulation in ITW children (Gwerder et al., 2025a; Assaiante et al., 2005). Existing literature presents conflicting findings. Rossi et al. (2018) observed varying H-reflex responses in ITW children in prone postures, with some showing increased, decreased, or even absent H-reflex activity compared to TD children. Children with ITW often exhibit sensory processing difficulties (Rossi et al., 2018; Williams et al., 2010), suggesting potential issues in the peripheral nervous system, which might explain the heterogeneity of H-reflex responses observed in the study from Rossi et al. (2018). These findings contrast with forward dynamic simulations that suggest increased plantarflexor reflex gains in ITW children and an altered neural control strategy similar to that of CP children (Veerkamp et al., 2023). These discrepancies may indicate that the central nervous system adjusts reflex gains dynamically to match functional demands. It would be important to ensure that such observations do not stem from methodological differences, and therefore studies including greater sample sizes are required in the future. Furthermore, while the inclusion of sensory mechanisms marks a significant step forward, the potential influence of cognitive and psychological factors that may also contribute to the observed differences, should not be overlooked.

### 4.4 Clinical perspectives

The findings of this study suggest that children with ITW and CP adopted distinct movement strategies in response to varying weight bearing conditions. CP children showed reduced gait variability and hyperreflexia, suggesting immature development of their sensorimotor systems. In contrast, children with ITW exhibited increased variability modulation and H-reflex responses comparable to those of TD peers, possibly indicating that these children take an atypical developmental path during early walking acquisition and altered sensorimotor adaptation. These patterns suggest an altered maturation of neuromotor control and spinal processing centers, highlighting the difficulty of compensating for underlying biomechanical constraints. Therefore, intervention strategies should aim to support the development and refinement of those motor strategies. Evidence suggests that reflex modulation can be learned, even in individuals with neurological disorders (Thompson and Wolpaw, 2021; Chen et al., 2005). Understanding the neuromotor etiology of toe walking lays the groundwork for developing more effective, individualized therapeutic approaches. Unlike Botulinum toxin A or other spasticity-reducing interventions, newer approaches aim to address the underlying causes more directly and with fewer side effects. Potential interventions could include spinal reflex conditioning (Thompson and Wolpaw, 2021), vibration therapy (Fanchiang et al., 2015), real-time feedback walking training (Cappellini et al., 2020; Brunner and Götz-Neumann, 2023), or enhancing sensory inputs through techniques such as stochastic resonance stimulation (Zarkou et al., 2018), plausibly addressing multiple systems simultaneously. However, further research is needed to refine these strategies and explore their long-term benefits.

### 4.5 Limitations

The study setup was complex, and several limitations should be acknowledged. The biggest limitation was the limited participation pool for ITW and CP children, potentially due to the restrictive eligibility criteria in order to include homogenous groups and to obtain relevant data. This study provides first evidence on how ITW and CP children adapt to different weight-bearing conditions and their underlying peripheral control mechanisms. However, larger studies are needed to confirm these findings. Additionally, due to the absence of ground reaction force data, the boundaries of the BoS were approximated using foot marker positions, which may have led to an overestimation of the MoS and did not account for medio-lateral ankle strategies. Furthermore, measurements were conducted on a treadmill at fixed speeds, which may not fully replicate the biomechanics of natural overground walking (Hollman et al., 2016). Walking speed usually changes under different weight-bearing conditions (Apte et al., 2018). However, instead of adjusting step width or walking speed, our participants may have used other strategies, such as foot placement or CoM regulation to adjust their gait patterns. Moreover, while the bodyweight support system effectively reduced loading, it also imposed movement constraints, particularly in the medio-lateral direction. This limitation may have restricted natural hip abduction and step width adjustments. Finally, the relatively short exposure time of 4 min per condition was intentionally chosen to minimize participant burden and avoid fatigue, particularly in children. While this duration allowed us to observe immediate responses to altered bodyweight, it may not have been sufficient to induce measurable gait or reflex adaptations beyond the initial learning phase. However, given our sample size and measurement setup, it is difficult to draw firm conclusions about such time-dependent adaptations. Future studies interested in long-term adaptations may therefore consider extending the duration or frequency of walking trials to better capture the evolution of motor adjustments under altered weight-bearing conditions. While this complex experimental setup may not have allowed complete movement freedom, it did allow for the capture of extended walking protocols.

## 5 Conclusion

A deeper understanding of the neuromotor etiology of toe walking under challenging conditions is crucial for developing targeted interventions. In this exploratory study, ITW children showed increased gait variability during bodyweight unloading, suggesting exploratory motor control under gait challenges. In contrast, CP children relied on a stiffening strategy, with unchanged variability and hyperreflexia, indicating limited capacity to explore alternative motor strategies, seemingly resulting in less efficient, compensatory gait patterns and reduced stability. These findings suggest that toe walking in both ITW and CP reflect distinctly different neuromotor adaptations to similar underlying biomechanical constraints, which should be confirmed in larger cohorts. A multifaceted approach targeting various sensorimotor and neuromotor control systems may hold the greatest promise for understanding gait stability, toward improving the overall quality of life in toe walking children.

## Data Availability

Due to ethical and privacy constraints, the data underlying this study cannot be shared publicly. It might be possible for the corresponding author to grant access to the datasets upon request and pending approval in accordance with ethical guidelines.
