# Supplementary material for: The effects of weight-bearing manipulations on gait and its underlying neural control mechanisms in toe walking children
Source: Front Hum Neurosci. 2025 Nov 10;19:1701454. doi: 10.3389/fnhum.2025.1701454 (PMC12640939; doi:10.3389/fnhum.2025.1701454)
Supplement: Supplementary file 1 [file Data_Sheet_1.pdf]

Supplementary Material

1 Marker Set

Name	Segment	Location
RTO1	Foot - right	Caput metatarsale I
RTO3	Foot - right	Metatarsale III
RTO5	Foot - right	Caput metatarsale VI
RFOX	Foot - right	Lateral, not specified (Extramarker)
RHEE	Foot - right	Calcaneus
RMMA	Shank - right	Medial malleolus
RLMA	Shank - right	Lateral malleolus
RTMT	Shank - right	unspecified Tibia, ventral
RTLF	Shank - right	unspecified Fibula, lateral
RTTT	Shank - right	Tuberositas tibea
RTIB	Shank - right	Caput fibulae
RMCO	Thigh - right	Medial femur epicondylus
RLCO	Thigh - right	Lateral femur epicondylus
RTFR	Thigh - right	unspecified Femur, front distal
RTLL	Thigh - right	unspecified Femur, lateral distal
RTLH	Thigh - right	unspecified Femur, lateral proximal
LTO1	Foot - left	Caput metatarsale I
LTO3	Foot - left	Metatarsale III

LTO5	Foot - left	Caput metatarsale VI
LHEE	Foot - left	Calcaneus
LMMA	Shank - left	Medial malleolus
LLMA	Shank - left	Lateral malleolus
LTMT	Shank - left	unspecified Tibia, ventral
LTLF	Shank - left	unspecified Fibula, lateral
LTTT	Shank - left	Tuberositas tibea
LTIB	Shank - left	Caput fibulae
LMCO	Thigh - left	Medial femur epicondylus
LLCO	Thigh - left	Lateral femur epicondylus
LTFR	Thigh - left	unspecified Femur, front distal
LTLL	Thigh - left	unspecified Femur, lateral distal
LTLH	Thigh - left	unspecified Femur, lateral proximal
RASI	Pelvis	Spina illiaca ant. sup. – right
RTMS	Pelvis	Iliaca – right
RPSI	Pelvis	Spina illiaca post. Sup – right
SACR	Pelvis	Sacrum
LPSI	Pelvis	Spina illiaca ant. sup. – left
LTMS	Pelvis	Iliaca - left
LASI	Pelvis	Spina illiaca post. Sup - left
RSHO	Shoulder	Acromion - right
MSTC	Shoulder	Manubrium sterni cranial

CVC7	Shoulder	C7
LSHO	Shoulder	Acromion - left
RWRA	Forearm - right	Proc. styloideus radii
RWUL	Forearm - right	Proc. styloideus ulnae
RFRA	Forearm - right	unspecified forearm, radial
RFUL	Forearm - right	unspecified forearm, ulnae
RMEC	Humerus - right	Medial epicondylus
RLEC	Humerus - right	Lateral epicondylus
RHVT	Humerus - right	unspecified upperarm, ventral
RHLT	Humerus - right	unspecified upperarm, lateral
LWRA	Forearm - left	Proc. styloideus radii
LWUL	Forearm - left	Proc. styloideus ulnae
LFRA	Forearm - left	unspecified forearm, radial
LFUL	Forearm - left	unspecified forearm, ulnae
LMEC	Humerus - left	Medial epicondylus
LLEC	Humerus - left	Lateral epicondylus
LHVT	Humerus – left	unspecified upperarm, ventral
LHLT	Humerus - left	unspecified upperarm, lateral
RFHD	Head	Headband
RBHD	Head	Headband
LFHD	Head	Headband
LBHD	Head	Headband

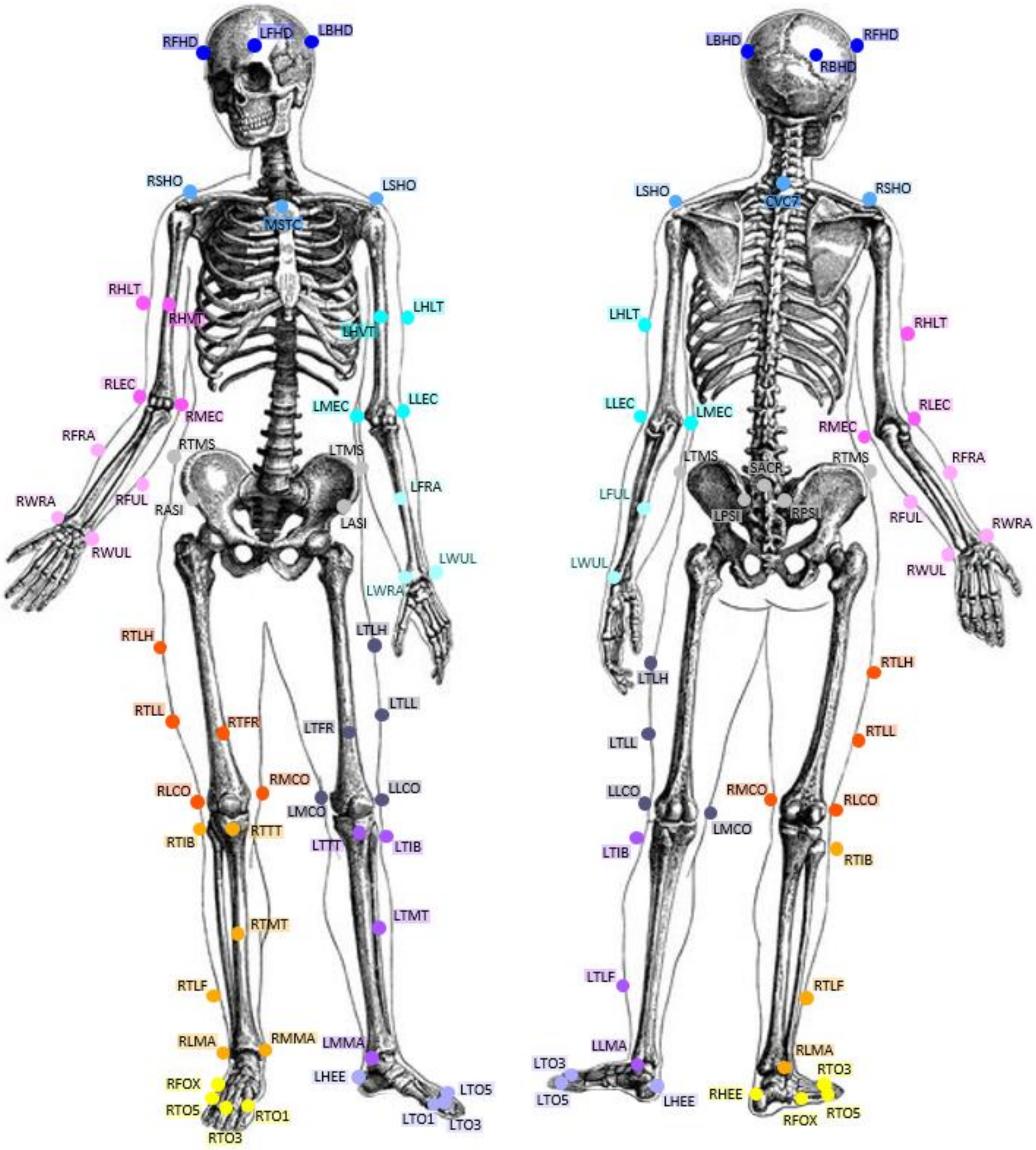

RFHD
LFHD
LBHD
RSHO
LSHO
MSTC
RHLT
RHVT
LHLT
LHVT
RLEC
RMEC
LMEC
LLEC
RFRA
RTMS
LTMS
LFRA
RWRA
RFUL
RASI
LASI
RWUL
LWUL
LWRA
RTLH
LTLH
RTLL
LTLL
RTFR
LTFR
RLCO
RMCO
LLCO
LMCO
RTIB
RTTT
LTTT
LTIB
RTMT
LTMT
RTLF
RLMA
RMMA
LMMA
RFOX
LHEE
LTO5
RTO5
RTO1
LTO1
LTO3
RTO3
LBHD
RFHD
RBHD
LSHO
CVC7
RSHO
LHLT
RHLT
LLEC
LMEC
RMEC
RLEC
LTMS
SACR
RTMS
RFRA
LFUL
LPSI
RPSI
RFUL
RWRA
LWUL
RWUL
LTLH
RTLH
RTLL
LTLL
RMCO
RLCO
LLCO
LMCO
LTIB
RTIB
LTLF
RTLF
LLMA
RLMA
LTO3
RTO3
RHEE
LTO5
LHEE
RFOX
RTO5

2 Statistical Analysis

Supplementary Material S1: Linear mixed-effects model results for mean gait parameters.

	Cadence	Stride Time	Single Support	Double Support	Stride Length	Step Width	MoS_{AP}	MoS_{ML}
**(Intercept)**	114.1325***	1.0556***	0.3975***	0.2559***	97.8157***	12.1869***	104.8249***	104.4298***
**GroupITW**	-1.2864	0.0328	-0.0036	0.0401	-18.8402	-0.3434	-15.4470	-11.0235
**GroupCP**	-15.6204	0.2300*	0.0258	0.1506*	-10.1189	0.1587	14.3044	13.1146
**BW070**	-0.3686	0.0182	0.0362***	-0.0423**	-0.0582	0.2895	-40.7917***	6.9677
**BW130**	2.3134	-0.0107	-0.0125	0.0142	-0.7209	-0.2109	-0.2855	1.4914
**GroupITW:BW070**	0.7003	0.0302	0.0092	0.0186	2.2852	0.3529	2.6858	3.7068
**GroupCP:BW070**	3.9149	-0.0101	-0.0048	-0.0152	0.4968	-0.8929	10.2400	-14.8687
**GroupITW:BW130**	-1.1736	0.0009	0.0049	-0.0067	0.3461	-0.0607	0.5807	-2.5985
**GroupCP:BW130**	-0.4048	0.0153	0.0150	-0.0096	-1.5427	-0.7379	-7.7385	-4.7424
**R^2**	0.87	0.95	0.87	0.93	0.96	0.53	0.77	0.63

Green positive and red negative correlation (* $p<0.05$, ** $p<0.01$, *** $p<0.001$). Typically developing group and BW100 were set as intercept. ITW: idiopathic toe walking; CP: cerebral palsy; MoS_{AP}: antero-posterior margin of stability; MoS_{ML}: medio-lateral margin of stability.

Supplementary Material S2: Linear mixed-effects model results for gait variability parameters.

	Stride Time	Single Support	Double Support	Stride Length	Step Width	MoS_{AP}	MoS_{ML}
**(Intercept)**	0.0422***	0.0176***	0.0289***	6.1175***	2.7100***	27.7955***	17.0225***
**GroupITW**	0.0138	0.0052	0.0115	0.0172	-0.1252	0.7138	1.8208
**GroupCP**	0.0170	0.0145	0.0140	0.1737	0.1986	5.6238	0.6859
**BW070**	0.0106	0.0088**	0.0075	0.9874	0.2483	1.9996	2.5912
**BW130**	-0.0026	-0.0017	-0.0016	-0.4544	0.5918	-3.0760	-0.0712
**GroupITW:BW070**	0.0219	0.0080	0.0128	1.9073	0.1483	14.3409*	3.0943
**GroupCP:BW070**	-0.0099	-0.0129*	-0.0030	-0.9653	-1.1786	-4.6523	-0.9862
**GroupITW:BW130**	0.0061	0.0039	0.0001	1.0068	-0.3258	5.3097	0.1051
**GroupCP:BW130**	0.0016	-0.0006	0.0015	0.1410	-0.9430	1.6634	-2.9566
**R^2**	0.80	0.74	0.86	0.68	0.46	0.70	0.75

Green positive and red negative correlation (* p<0.05, ** p<0.01, *** p<0.001). Typically developing group and BW100 were set as intercept. ITW: idiopathic toe walking; CP: cerebral palsy; MoS_{AP}: antero-posterior margin of stability; MoS_{ML}: medio-lateral margin of stability.

Supplementary Material S3: Linear mixed-effects model results for reflex parameters

	**H-reflex**	**H-reflex$_{norm}$**	**bEMG**
**(Intercept)**	2.6558***	49.6860***	0.0813***
**GroupITW**	-0.4172	-7.8159	-0.0165
**GroupCP**	0.5956	57.7675***	-0.0481*
**BW070**	-1.0792***	4.7676	-0.0352*
**BW130**	0.0221	-5.4354	0.0054
**GroupITW:BW070**	0.5825	-1.5881	0.0109
**GroupCP:BW070**	0.1412	7.0907	0.0248
**GroupITW:BW130**	0.3937	13.2608	-0.0069
**GroupCP:BW130**	-0.4707	12.0378	-0.0040
**R^2**	0.71	0.63	0.39

Green positive and red negative correlation (* p<0.05, ** p<0.01, *** p<0.001)). Typically developing group and BW100 were set as intercept. ITW: idiopathic toe walking; CP: cerebral palsy; H-reflex$_{norm}$: H-reflex normalized to background EMG (bEMG).
